# Life history and ecology shape global patterns in avian hatching failure rates

**DOI:** 10.1186/s12862-026-02515-x

**Published:** 2026-04-10

**Authors:** Oddvar Heggøy, Jostein Gohli, Helene Dale Johnsen, Terje Lislevand

**Affiliations:** 1 Department of Natural History, University Museum of Bergen, University of Bergen, PO Box 7800, Bergen, NO-5020 Norway; 2 Norwegian Institute for Nature Research, Sognsveien 68, Oslo, NO-0855 Norway

**Keywords:** Bird eggs, Clutch size, Latitudinal trends, Hatching failure, Life history, Longevity, Reproduction

## Abstract

**Background:**

Hatching failure is common in the eggs of wild birds, yet its variation in relation to ecological and life history factors remains poorly understood. Understanding these patterns is essential for explaining reproductive strategies and evolutionary trade-offs in birds.

**Results:**

In a comprehensive comparative study, we found that the previously recognized latitudinal decline in avian hatching failure rates is driven by a greater proportion of eggs failing in larger clutches at lower latitudes. We suggest that this pattern could reflect higher exposure to ambient conditions in larger clutches, which might have more severe consequences for egg viability in warmer environments. Furthermore, hatching failure rates increased with species longevity. We predicted this from the notion that, if needed, short-lived species should invest more heavily in current reproduction, whereas longer-lived species should be more prone to prioritize self-maintenance to improve future breeding opportunities. Hatching failure rates were also higher in species with uniparental incubation compared to biparental species, higher in elevated nests than in ground nests, and negatively related to altitude.

**Conclusions:**

Our results reveal several novel patterns of variation in hatching failure rates in relation to aspects of ecology and life history. These results suggest that clutch size, latitude, and parental care strategies interact to shape reproductive success and highlight potential evolutionary trade-offs between current and future reproduction. Further research is needed to clarify the proximate mechanisms underlying these patterns and their implications for avian life history evolution.

**Supplementary Information:**

The online version contains supplementary material available at 10.1186/s12862-026-02515-x.

## Background

Hatching success is an integral part of fitness in birds, and successful embryo development depends on maintaining eggs within a suitable thermal range during both the laying and incubation periods [1]. The temperature range for optimal development of bird embryos is typically 36–39 °C [1, 2]. Prolonged exposure to temperatures outside the optimal range, whether too warm or too cold, may negatively affect embryo development or cause hatching failure [1–3]. However, bird embryos are typically more tolerant to periods of hypothermia than to hyperthermia [1, 4]. If temperatures fall below the “physiological zero”, which is generally considered to be approximately 24–26 °C [1, 2], embryo development is arrested. Egg temperatures are influenced by several ecological and behavioural variables, such as parental incubation strategy and nest attentiveness [1–3], clutch size [5, 6], egg size [7], nest structure [1, 5], nest placement [3, 8], and ambient environmental conditions [9–12]. Many of these thermal determinants vary geographically. Most importantly, ambient temperatures decline with increasing latitude and altitude. Clutch size shows a well-established increase toward higher latitudes [10, 13, 14], and often declines with altitude [15, 16]. In addition, nest attentiveness generally increases with latitude [17], while parental care strategies may vary both across latitude and altitude [13, 17, 18]. These ecological and behavioural variations may therefore generate broad-scale geographical variation in the thermal conditions that developing embryos experience.

Hatching failure is widespread in birds. On average, approximately 10–15% of bird eggs remaining in the nest at the end of the incubation period fail to hatch, with considerable variation among species [19, 20]. These unhatched eggs are often regarded as unfertilized, but several studies indicate that embryos may be present in most cases [21–24]. Although elevated rates of hatching failure in birds could be related to anthropogenic environmental changes [19, 21], there is also evidence that hatching failure occurs naturally and varies in relation to both intrinsic (physiological, life history) and extrinsic (ecological, environmental) factors [5, 20].

In a seminal comparative study, Koenig [20] reported that avian hatching failure rates were negatively correlated with latitude. However, the reason for this relationship is still unclear. Here, we hypothesize that this strong latitudinal correlation reflects a temperature-dependent effect of clutch size (hereafter “*the clutch size hypothesis*”). The key factor behind this pattern could be geographically variable exposure of eggs to suboptimal incubation conditions which might result from at least two non-exclusive mechanisms. First, because many birds initiate stable incubation only when approaching clutch completion and most birds lay a maximum of one egg per day [1], eggs laid early in the laying sequence in large clutches can be exposed to ambient temperatures for several days [25, 26]. In warm environments as is typically found at lower latitudes, there is a higher risk of exposure to temperatures above the physiological zero before the onset of stable incubation, which may cause spontaneous and irregular embryo development [2, 25–27]. Second, parents’ capacity to maintain optimal incubation temperatures may be reduced in large clutches [28], and clutch size could therefore affect their ability to prevent eggs from overheating under warm conditions. In addition, larger clutches might show higher thermal inertia [3] and thus lower heat loss rates. In both cases, embryo survival might be compromised, causing higher hatching failure rates in warmer environments. In contrast, these effects may be of less importance in the cooler conditions typical of higher latitudes.

We further propose that interspecific variation in hatching failure may also reflect long-recognized life history trade-offs. For parent birds, incubation is costly because of direct energetic demands [29, 30], thermoregulatory challenges [31], and increased predation risks [26, 32–34]. Accordingly, parent birds must balance current reproductive investment against their own requirements for self-maintenance and future survival [32, 35–37]. If needed, parents in longer-lived species should therefore be more prone to prioritizing self-maintenance over high incubation efforts to avoid compromising future reproductive opportunities [36, 37]. If prioritizing self-maintenance results in lower nest attentiveness or otherwise reduced incubation efficiency, hatching failure rates should be higher in long-lived species, whereas short-lived species are expected to invest more heavily in current reproduction and have lower hatching failure rates (hereafter *“the longevity hypothesis*”).

Previous studies suggested that several other ecological and life history factors might also affect hatching failure rates through their effects on incubation temperatures. First, species with uniparental incubation generally have lower nest attentiveness than biparental incubators [1], which results in higher exposure of eggs to ambient conditions and potentially higher hatching failure rates in the former group [20]. Second, open nests have been reported to show higher hatching failure rates than closed ones, presumably due to differences in nest microclimate [5], and nests above the ground may be more exposed to convective cooling or heating than ground nests [8]. Third, as larger eggs have a lower surface-to-volume ratio than smaller eggs, thereby reducing heat transfer between eggs and the environment, embryos may be less vulnerable to ambient temperature fluctuations in larger eggs [7]. Finally, although hatching failure rates in birds are not known to vary with altitude [20, 38, 39], it is still possible that altitudinal effects vary in magnitude across a latitudinal gradient, as reported for other avian life history variables like clutch size, egg size, and extra-pair paternity [16, 40].

We carried out a comparative study to investigate possible ways in which hatching failure rates in birds could be related to aspects of ecology and life history. From the clutch size hypothesis, we predicted that hatching failure rates should increase with clutch size in birds nesting in warm environments at low latitudes, but not elsewhere. Moreover, as predicted by the longevity hypothesis, hatching failure rates should be higher in longer-lived species. From previous reports on the influence of other ecological factors, we also predicted higher rates of hatching failure (1) in species with uniparental rather than biparental incubation [1, 20], (2) in species with open rather than closed nests [5], (3) in species nesting above ground rather than on the ground [8], and (4) in species laying smaller eggs [7]. Finally, if the risk of hatching failure is highest in warm climates at low latitudes, it might be reduced in alpine habitats where temperatures are presumably lower and less harmful to bird embryos [38, 39].

## Methods

### Dataset

In accordance with the calculation of “hatchability” by Koenig [20] and more recent comparative studies of this phenomenon [19], we here define hatching failure rate as the proportion of all eggs in a clutch present at the end of the incubation period that never hatches. Consequently, we excluded cases (eggs, nests, and studies) where hatching failure resulted from predation, nest abandonment, physical damage to eggs caused by observer handling, or human disturbance of the nest. We also excluded cases where the fate of eggs was unclear, but such cases were rare in the assembled dataset, as most studies reported hatching outcomes explicitly. Noteworthy, our measure of hatching failure reflects mean values across clutches and does not capture the distribution of hatching failure among individual nests (e.g., consistently low vs. occasionally high failure rates), which may vary in relation to parental quality and age.

We collated estimated hatching failure rates in birds or data that could be used to calculate hatching failure rates from various sources. First, we searched for data in previously published comparative studies of hatching failure [5, 19, 20, 41–45]. Most of these data are publicly available, but the data from Koenig [20] were provided by the author. We checked the values of these hatching failure rates in primary sources to determine whether the calculations matched our definition of the variable and excluded them if they failed to do so (*n* = 110 estimates). Most data from Møller et al. [45] were excluded for this reason. Nevertheless, the information available in the primary sources of that paper often made it possible to recalculate hatching failure rates according to our standards. Overall, 452 estimates of hatching failure rates from 306 species were included from previously published comparative work. In addition, we searched Google Scholar and ISI Web of Science for relevant information using the terms “hatching failure”, “hatching success”, “hatchability”, “birds”, and “breeding ecology”. This literature search added 416 estimates from 204 different species to the dataset. Finally, we extracted data from the nest records archive at the University Museum of Bergen, Norway (solely from Norwegian breeding birds; *n* = 31 species, of which 11 were not in the dataset before).

As we here focus on possible natural causes of hatching failure, we do not include data from (1) eggs incubated artificially or laid by birds kept or bred in captivity, (2) eggs from populations that were supplementally fed, (3) experimentally manipulated birds or clutches, (4) populations of common species known to be highly inbred, and (5) eggs from sites that were known to be severely contaminated by pollutants. Furthermore, brood parasitism (by, e.g., cuckoos (Cuculidae) and cowbirds (Icteridae)) is a natural factor that might affect hatching failure rates. However, since our dataset includes only a few such cases (*n* = 18 estimates from 4 species) and since their breeding ecology is unusual and unique, we excluded hatching failure rates from both the brood parasites themselves and the host’s eggs in parasitized nests. For consistency, we also omitted eggs that were certainly replacement clutches, although such information was often not reported in the data sources. In total, fewer than 20 estimates were excluded for this reason and any potential bias introduced by this exclusion should therefore be minimal. Finally, we omitted studies based on < 25 eggs and/or < 10 clutches (in accordance with Koenig [20]). By applying these thresholds, we reduced the risk that estimates were randomly skewed due to small sample sizes while still preserving broad taxonomic and geographical representation in the dataset. Throughout the text, the common and scientific names of bird species followed the IOC World Bird List taxonomy [46].

All recorded variables are summarized in Table 1, along with predictions for the interspecific variation in avian hatching failure rates. As a proxy for lifetime expectancy in birds, we used species-specific estimates of Generation Length [47]. The variables Nest Type, Nest Site and Incubation Strategy are species specific, and data for these variables were primarily found in ornithological handbooks such as Cramp et al. [48] and Birds of the World [49]. When available, we used information on these variables provided in the primary sources of hatching failure rates. We classified the Nest Type as “closed” if the nest usually has a structure covering the eggs from above (made by the birds or natural cavities) and “open” if such cover is not present. The Nest Site was classified as (primarily) “on ground” (including cliff walls or, more rarely, floating on water) or (primarily) “above ground level” (elevated) in grass, shrub, or in a tree. Moreover, we classified Incubation Strategy as “uniparental” (incubated by one parent) or “biparental” (incubated by both parents) and used this variable as a proxy for nest attentiveness [1], allowing us to explore broad-scale patterns in the absence of more detailed behavioural data from the incubation period in many species. We specifically tested for a phylogenetic signal in hatching failure rates in the present study (see below), and the variable “phylogenetic order” (following Gill et al. [46]) was therefore included only to illustrate the distribution of estimates across orders (Fig. S1).

**Table 1:** Explanatory variables included in the study, along with predictions for the interspecific variation in avian hatching failure rates and the background (with relevant sources) for these predictions

Variable	Background	Predicted direction of hatching failure rates
*Latitude*	Eggs are more exposed to suboptimal and detrimental environmental conditions (e.g., higher temperatures) at lower than at higher latitudes [25–27, 77]	Declining with latitude
*Altitude*	Offspring quality increases with altitude [16, 18], higher and potentially more harmful ambient temperatures at lower altitudes [25–27, 77]	Declining with altitude
*Latitude x* *Altitude*	The decline in hatching failure rate at lower latitudes should be modified by altitude, by e.g. declining environmental temperatures at higher elevation [25, 26]	Lower at high altitudes at low latitudes, no effect at high latitudes
*Clutch Size*	Larger clutches suffer from higher environmental exposure due to delayed incubation onset [25–27] or limits to incubation capacity [5, 28]	Increasing with clutch size
*Clutch Size x Latitude*	Higher environmental exposure in large clutches (see above) may be more problematic in the warm environments at low latitudes.	Increasing with clutch size at declining latitudes
*Incubation Strategy*	Clutches of species with uniparental incubation generally experience lower nest attentiveness and may suffer from higher environmental exposure than clutches of species with biparental incubation [1].	Lower in species with biparental incubation
*Egg Size*	Larger eggs have lower surface to volume ratio, and may be less vulnerable to variable ambient temperatures [78, 79]	Declining with egg size
*Generation length*	Short-lived species should invest relatively more in current reproduction than longer-lived species due to different probabilities of breeding again in the future [36, 37, 28]	Increasing with generation length
*Nest Type*	Closed nests often have larger clutches, which may suffer from higher environmental exposure due to delayed incubation onset or poor incubation behaviour [5, 20]	Higher in closed than open nests
*Nest Site*	Ground nests are less affected by thermal convection than nests above the ground [8]	Higher in nests above than on the ground
*Nest Type x* *Nest Site*	Less thermal convection in closed nests can modify effects of nest site (see above) [5, 6, 20]	Higher in open nests above ground

Species means of Clutch Size and Egg Size were extracted from Lislevand et al. [50], from textbook descriptions or from primary sources on hatching failure rates if this information was provided. We used fresh egg mass as a measure of egg size, as this value is available for a larger number of species than (the cube root of) average egg volume, which was used by Koenig [20].

The geographical position (Latitude and Altitude) of each study site was extracted from information given in the original sources of hatching failure data or, more rarely, from secondary sources if not available in the original descriptions. We were interested in the effect of distance to the equator, irrespective of hemisphere, and therefore used absolute values of latitude in the analyses. For each location, we sampled altitude data from Google Earth. If studies were performed at several locations, we used the mean latitude and altitude. If data were reported from a larger region (e.g., national schemes), we used the approximate geographical midpoint of that region. In a few cases, the positions of the study sites were inaccurately reported, and if our estimate of altitude from these sites exceeded the elevational ranges reported for the species by the IUCN [51], we corrected the estimate to the approximate midpoint of these species-specific elevational ranges. When more than one estimate was available per species, we calculated the mean species-specific values of the hatching failure rate and explanatory variables for use in further analyses.

### Analyses

All the statistical tests were performed in R version 4.1.1 [52]. To control for phylogenetic relationships between species [53], we matched our dataset with the nomenclature of Jetz et al. [54] (using the website www.birdtree.org) to produce a subset of 10,000 phylogenetic trees on the basis of the backbone phylogeny of Hackett et al. [55] and the matching species (489 out of 521 species) from our dataset. We summarized the information from the phylogenetic trees into a single consensus tree (see Heggøy et al. [56]) with TreeAnnotator from the program BEAST 2 2.7.1 [57]. We used the R package “SLOUCH” to test for phylogenetic signal in hatching failure rates. This program fits an “optimal regression model” of evolution based on an Ornstein–Uhlenbeck model, described in detail by Hansen et al. [58]. The model estimates adaptation in the evolution of a trait along a phylogenetic tree and simultaneously controls for phylogenetic inertia. This differs from many other methods that account for phylogenetic correlations between species, as these methods typically do not account for such constraints (inertia) [58, 59]. A half-life parameter (*t*_1/2_), usually interpreted as the time it takes for a trait to evolve half the distance between the ancestral state and the predicted optimum, indicates whether there is any evolutionary lag in the adaptation of the trait. If the half-life equals zero, there is no such lag, whereas a half-life above zero indicates a phylogenetic constraint on adaptation of the trait towards the optimal state. Evolutionary changes in the response variable caused by genetic drift and other unmeasured selective forces are expressed by the stochastic stationary variance (v_y_) of the model. The half-life parameter from the evolutionary regression model in SLOUCH provides a measure that corresponds to the distance to the root of the phylogeny. This makes it more biologically interpretable and scalable than the more widely used Pagel’s lambda (λ), which measures the extent to which a trait’s distribution conforms to Brownian motion [58–60]. In the present study, we fitted an intercept-only model without predictors to measure the phylogenetic signal associated with the hatching failure rate (i.e., how much of the variation in hatching failure is explained by phylogeny). The total length of the phylogeny was scaled to 1 for easier interpretation of the half-life (phylogenetic signal).

The phylogenetic signal in our dataset was negligible (see results), implying that there was no need to use phylogenetically controlled methods to analyse our data. Notably, this differs from another recent analysis of avian hatching failure rates, which reported a stronger phylogenetic signal in their data [19]. This is likely explained by differences between the two studies in the methods used to test for phylogenetic signal, and in the data and sample sizes. Since hatching failure rate values are proportions showing a heavily left-skewed distribution, we used beta regression (R package “betareg”, which fits regression models to beta distributed data using maximum likelihood; [61]) to analyse our data.

We built multivariate models including all possible combinations of our predictor variables and evaluated model performance using Akaike Information Criterion (AIC) scores. All models within 2 ΔAIC of the top model (the one with the lowest AIC score) were considered competitive [62]. We evaluated competitive models for uninformative parameters [63] and chose the most parsimonious alternative as our best supported model. We considered parameter estimates with 85% confidence intervals overlapping zero as uninformative to ensure consistency between AIC model selection and parameter evaluation criteria and therefore report 85% intervals along with the conventional 95% intervals [63, 64].

Multivariate models included the hatching failure rate as a response variable and Latitude, Altitude, Clutch Size, Egg Size, Incubation Strategy, Generation Length, Nest Type and Nest Site as explanatory variables. To test the prediction that hatching failure rates increase more with clutch size at low latitudes (Table 1), we included an interaction between Clutch Size and Latitude. The prediction that altitudinal effects vary in magnitude across latitudes was tested by including an interaction between Altitude and Latitude (Table 1). Finally, the candidate models included an interaction between Nest Type and Nest Site, as each of these variables could be expected to influence the microclimatic effects of the other variables (Table 1). Before fitting the models, we examined all predictors for collinearity. Egg Size was positively correlated with Generation Length (*r* = 0.54; Fig. S2), and for some of the species in our dataset (*n* = 27), we could not retrieve data on this parameter. As this could influence our results, we built models both with and without Egg Size as a predictor. We also considered Developmental Mode (altricial or precocial) as a potential predictor, given its association with several life history traits and potential influence on nest conditions. However, the variable was strongly correlated with Nest Site (*r* = 0.58) and showed no effect in model sets where it replaced Nest Site. It was therefore excluded from further tests. The best supported model(s) had some influential observations (Cook’s distance > 4/*n*, *n* = 27), and this problem was not solved by transformation of any of the variables. We therefore built additional models without these highly influential points to check whether this affected any of our conclusions.

## Results

### The dataset

The full dataset contained estimates of hatching failure from 521 different species and 26 phylogenetic orders of birds (Fig. S1). Information about all explanatory variables included in multivariate analyses was available for 474 of the species, and for 496 species if Egg Size was excluded (Table S1). Studies from the Northern Hemisphere (82.3%) and North American (41.8%) and European (35.8%) countries made up most of the dataset (Fig. S3). Hatching failure rates ranged from 0% to 60% (median = 8%, quartiles = 6% and 13%, *n* = 521). A previous analysis revealed that there were no clear temporal trends in hatching failure rates over the last century in our dataset [56].

### Phylogenetic tests

The intercept-only model from the phylogenetic test produced a very low half-life value (*t*_1/2_ = 1 * 10^− 8^ after the phylogeny was scaled to a total length of 1; see Heggøy et al. [56]). This suggests that hatching failure rates are not phylogenetically structured in our dataset. Additionally, univariate adaptation models for the explanatory variables produced low half-life values (*t*_1/2_ < 0.002 in all cases), indicating that there were no phylogenetic constraints on the evolution of hatching failure rates.

### Model selection outcomes

In model sets excluding Egg Size, all five competitive models included additive effects of Generation Length, Nest Site, Incubation Strategy, Altitude and the interaction between Latitude and Clutch Size (Table S2). There was only a marginal effect of Altitude in the best supported model (Table 2), but the effect of this variable was more pronounced (estimate and CIs per 100 m elevation: β = −0.015, 95% CI: -0.0288, -0.0020, 85% CI: -0.0252, -0.0056) after the removal of some influential points (Table S3). Except for this, the removal of outliers did not change any of our conclusions. We found no effect of Egg Size in model sets including this variable (model-averaged coefficients: β = −0.0008, 95% CI: -0.0024, 0.0009, 85% CI: -0.0019, 0.0004). Since the inclusion of Egg Size reduced the number of species represented in our dataset, we hereafter focus solely on the best supported model from model sets excluding Egg Size as an explanatory variable.

**Table 2:** Parameter estimates and confidence intervals (CI; 95% and 85%) from the best supported multivariate beta regression model explaining hatching failure rates (*n* = 496) with Generation Length [51], Clutch Size, Incubation Strategy, Latitude (absolute values), Nest Site and Nest Type, and interactions between Latitude and Clutch Size

Variable^1^		95% CI		85% CI	
	Estimate	lower	upper	lower	upper
(Intercept)	-3.664	-4.325	-3.003	-4.150	-3.178
**Main effects**					
Generation Length	0.062	0.045	0.079	0.049	0.075
Clutch Size	0.439	0.215	0.663	0.274	0.603
Latitude	0.064	0.033	0.095	0.041	0.087
Latitude^2^	-0.0007	-0.001	-0.0003	-0.001	-0.0004
Altitude (per 100 m)	-0.012	-0.025	0.00028	-0.022	-0.0031
Nest Site (ground vs. elevated)	-0.239	-0.039	-0.092	-0.347	-0.131
Incubation (bi- vs. uniparental)	-0.189	-0.338	-0.039	-0.298	-0.079
**Interaction effects**					
Latitude : Clutch Size	-0.015	-0.025	-0.005	-0.022	-0.008
Latitude^2^ : Clutch Size	0.00013	0.00002	0.00024	0.00052	0.00021

^1^: Variables and interactions not included in the final model: Nest Type, Latitude: Altitude, Nest Site: Nest Type

**Fig. 1:**
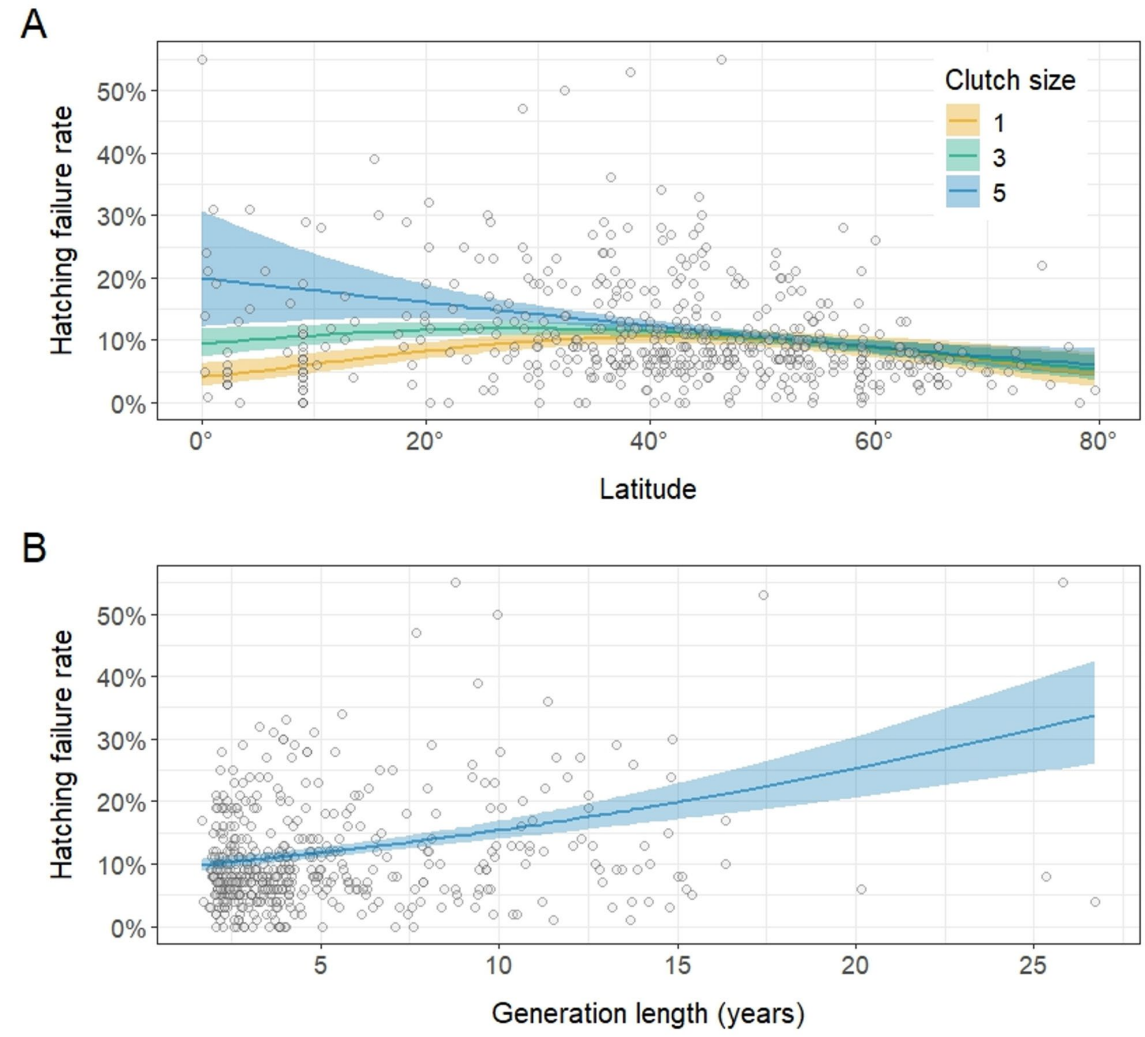
Distribution of hatching failure rates in birds (raw data points, *n* = 496) in relation to **A** Latitude (absolute values) of the study site and **B** Generation Length [49], with model predictions (regression lines with 95% confidence bands). In plot (a), regression lines visualize the interaction between Latitude and Clutch Size, with colours corresponding to different clutch sizes (predefined: 1 egg – yellow, 3 eggs – green, 5 eggs – blue). Effect sizes are from the beta regression model shown in Table 2

Hatching failure rates were highest at low latitudes and declined towards higher latitudes (Table 2; Fig. 1A), but the latitudinal effect was dependent on Clutch Size. Specifically, at low latitudes, species with large clutch sizes had higher hatching failure rates than did species with smaller clutch sizes, but an effect of Clutch Size was not evident at higher latitudes (Table 2; Fig. 1A). Hatching failure rates were also negatively related to Altitude in our dataset, but contrary to our predictions, this effect did not depend on Latitude. Furthermore, as predicted, we found a positive relationship between hatching failure rate and Generation Length (Table 2; Fig. 1B).

**Fig. 2:**
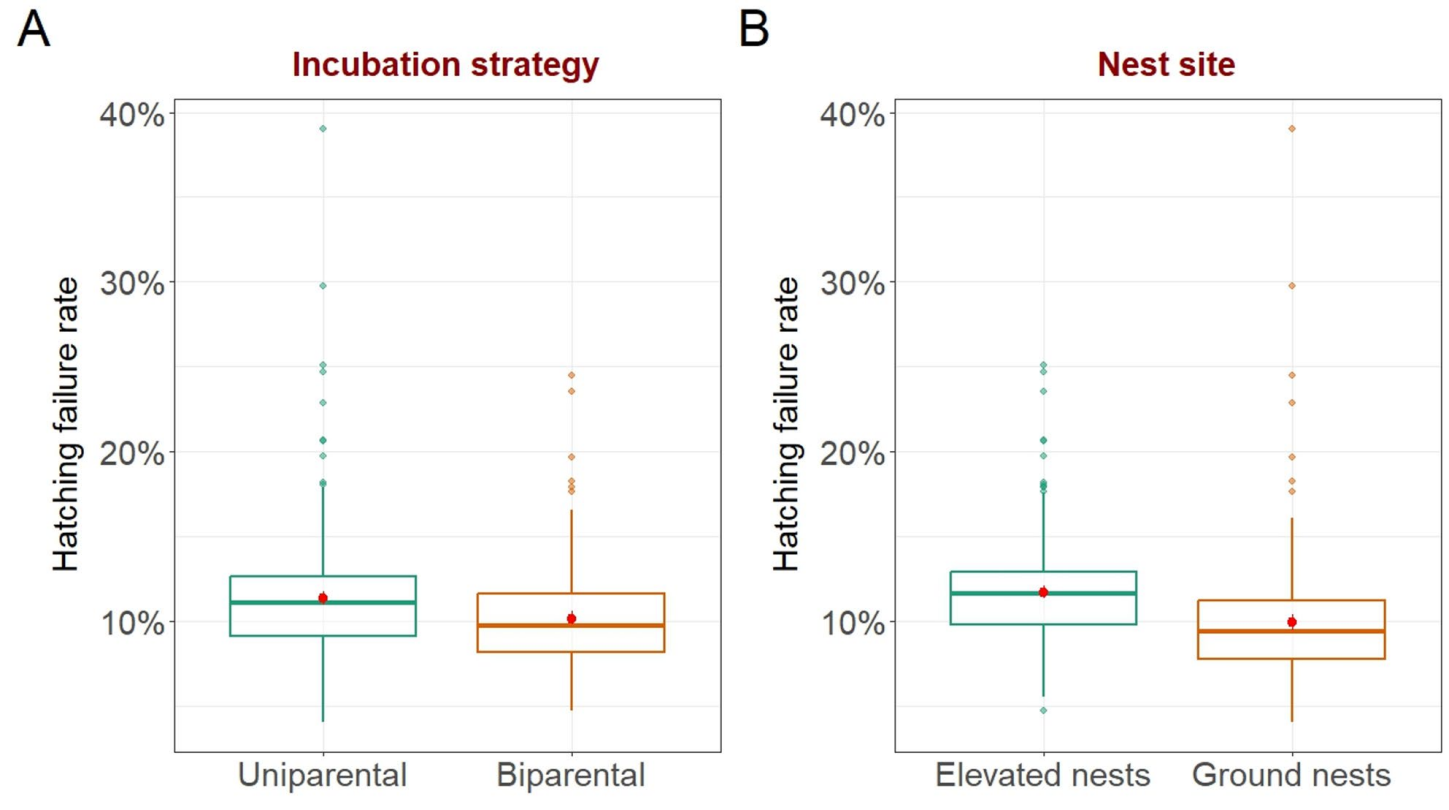
Hatching failure rates of birds (*n* = 496) in relation to the **A** Incubation Strategy and **B** Nest Site. Box plots show effect sizes from the beta regression model shown in Table 2, with median predicted values (horizontal line within boxes), interquartile ranges (IQRs; boxes) and variability outside the upper and lower quartiles (1.5 times the IQR). Red dots indicate bootstrapped mean estimates per category

Species with uniparental incubation had higher hatching failure rates than those with biparental incubation (predicted values (95% CI): uniparental: 13% (11–14%), biparental: 11% (10–12%); Table 2; Fig. 2A). Hatching failure rates were also higher in species nesting above ground level than for those nesting on the ground (predicted values (95% CI): elevated nests: 13% (12–14%), ground nests: 10% (9–11%); Table 2; Fig. 2B). Nest Type was not retained in the best supported model, although some candidate models indicated a marginal effect of this variable (Table S2), with higher hatching failure rates in species breeding in closed nests than in those breeding in open nests. There was no effect of the interaction between Nest Site and Nest Type, meaning that nests above ground level had higher rates of hatching failure than ground nests did, irrespective of whether they were open or closed. To further explore why nests placed above the ground had higher hatching failure rates, we checked whether the clutch sizes were larger in these nests. However, no difference in Clutch Size was found between ground-nesting and above-ground nesting birds (medians: ground nests: 3.8, elevated nests: 3.5; Wilcoxon test; *p* = 0.72, effect size *r* = 0.02, *n* = 515) or between species with open nests and those with closed nests (medians: open nests: 3.7, closed nests: 3.7; Wilcoxon test; *p* = 0.63, effect size *r* = 0.02, *n* = 515).

## Discussion

Based on a global dataset including more than 500 species of wild birds, our study provides a broad assessment of avian hatching rate ecology. Our analyses revealed several novel patterns, namely, that hatching failure rates (1) increase with clutch size at low but not high latitudes, (2) increase with species longevity, (3) are higher in nests placed above than on the ground, and (4) generally decrease with altitude. We suggest that all these patterns may be influenced in part by variable thermal conditions in the nest.

The first key finding in our study clarifies the long-standing observation that avian hatching failure rates decline with latitude [20] by showing that this pattern is driven primarily by higher failure rates in large clutches at low latitudes, as predicted by our clutch size hypothesis (Table 1). This might be explained by increasing risk of exposure of embryos to detrimental temperatures in large clutches in warmer regions of the globe. Indeed, empirical evidence shows that eggs at low latitudes are more likely to experience temperatures above the “physiological zero” [9–11], potentially triggering irregular embryo development before stable incubation commences [1, 2]. Exposure to lethal heat should also be more likely at low latitudes [10, 12], although some species may mitigate this by breeding during less warm periods of the year [9]. Bird eggs are generally tolerant of low temperatures [1, 4, 38], but prolonged exposure to sub-optimal and low temperatures can still lead to a range of detrimental effects on developing offspring, both before and after hatching [3]. However, parents may be more able to protect their eggs against cold than warm ambient temperatures and preventing overheating may impose higher behavioural and physiological costs [2, 65, 66].

We envisioned two non-exclusive reasons why hatching failure rates should co-vary with Latitude and Clutch Size as described above. First, *the egg viability hypothesis* predicts that eggs in larger clutches are exposed to ambient conditions for a longer time if incubation onset is delayed until clutch completion, leading to higher hatching failure risk in early-laid eggs, especially in warm environments [25, 26]. Several species-specific studies support this hypothesis, and some reported higher hatching failure rates in larger clutches at low latitudes [9, 10, 27] in line with the interaction between Clutch Size and Latitude in the present study. Noteworthy, however, many birds also initiate incubation before clutch completion [67], with considerable variation between species, populations and individuals [1]. The relevance of the egg viability hypothesis may therefore vary accordingly. Second, *the incubation limitation hypothesis* suggests that exposure of eggs to ambient conditions increases with clutch size due to limited incubation capacity of parents [28]. Support for this idea comes from studies showing generally lower and more variable egg temperatures in larger clutches [5, 6]. Higher energy demands associated with incubating more eggs may also sometimes force parent birds to spend more time foraging, thereby leading to reduced nest attentiveness [68], which may further compromise incubation conditions. Clutch size generally decreases towards the equator [13, 14, this study], a pattern traditionally attributed to variation in resource availability and predation risk [13, 69, 70]. Thermal constraints on egg viability represents an additional mechanism that may select for smaller clutches at low latitudes [9, 25–27].

Several alternative explanations for the interaction between Clutch Size and Latitude are possible, such as the depletion of maternal resources over the course of the egg laying period [35, 71], which could reduce egg quality and increase hatching failure rates in late-laid eggs. If resource availability increases at higher latitudes due to longer summer days [28, 72], resource depletion may be more pronounced in large clutches at low latitudes. Another factor that might be relevant here is the infestation rates of parasites and microbes which also increases towards the tropics [73] and could negatively affect hatching rates [38]. If infestation rates increase with environmental exposure of eggs during laying, this problem may be more acute in larger clutches [38]. Finally, one could hypothesize that parents may be more inclined to reduce their incubation efforts in a large clutch under challenging conditions (e.g., high temperatures, poor body condition, etc.), as the cost of losing an egg represents a smaller proportional reduction in reproductive output than in a small clutch. To better understand all these possible dynamics in hatching failure ecology, future work is needed to more directly test the effects of e.g., laying chronology, egg temperature, and clutch size on avian hatching failure rates [9] via detailed data collected across wide latitudinal gradients and from a diversity of species.

As predicted from the longevity hypothesis, we also found a strong positive relationship between avian hatching failure rate and Generation Length. This hypothesis rests on the idea that, if needed, species with longer life expectancies should more often than short-lived species prioritize self-maintenance and future reproductive opportunities over current reproduction (Table 1) [36, 37, 74]. Several studies have confirmed a trade-off between hatching success and self-maintenance within species [e.g., 7, 75], especially in parents with poor body condition, which presumably reflects birds’ inability to incubate their eggs efficiently [76]. However, alternative explanations for our result might be suggested. First, longevity or mortality risk *per se* could influence hatching failure risks through variation in avian incubation behaviour, as birds with low levels of adult mortality have been found to show reduced nest attentiveness [74, 77, 78]. Moreover, an underlying variable that is positively correlated with Generation Length could perhaps have resulted in false support for the longevity hypothesis. One such possible variable is incubation period which generally increases with avian life expectancy [74, 78]. Long incubation periods might increase the risk of egg mortality simply by chance because eggs are exposed to any detrimental factors for a longer time [74, 79]. Furthermore, both incubation period and adult mortality risk are sometimes shown to be negatively correlated with egg temperature, reflecting lower nest attentiveness and possibly less parental investment as incubation periods increase [77, 78, 80]. Conversely, it has also been suggested that longer incubation periods could be associated with higher parental investment in offspring due to a trade-off between embryo growth rate and offspring quality [77, 81]. In such cases, a positive relationship between the duration of the incubation period and hatching success would be expected. Among the few studies investigating these questions, however, there is little evidence for a relationship between the duration of incubation periods and hatching failure rates [5, 82].

Uniparental species in our dataset had higher hatching failure rates than bi-parental species, consistent with reduced nest attentiveness in species with uniparental incubation [20]. Interestingly, nest attentiveness and the frequency of biparental care tend to decrease at lower latitudes [17, 77], which should lead to increased exposure of eggs to environmental conditions [2]. However, nest attentiveness could not be evaluated directly in the present study. We also had a relatively low number of tropical species in our dataset (Fig. 1A) which makes it difficult to compare incubation in these birds with those at higher latitudes. The incorporation of a broader representation of tropical and other taxa, along with reliable metrics of nest attentiveness and hatching rates, could provide a deeper insight into how these factors are inter-related.

Hatching failure rates were higher in species which built their nests in elevated positions in shrubs and trees compared with species building nests on the ground. Eggs in nests situated above the ground may experience other thermal conditions than eggs on the ground. For example, Zerba and Morton [8] reported lower incubation temperatures in elevated nests than in ground nests of white-crowned sparrows *Zonotrichia leucophrys*. This was suggested to be caused by more rapid convective cooling in nests located above the ground. As should be expected, these authors also reported a considerable difference in hatching failure rates between elevated nests (9%) and ground nests (5%) [8]. Our dataset did not allow us to test specifically for microclimatic effects in individual nests, and this question needs more detailed investigation. Furthermore, possible differences in predation risk between ground nests and elevated nests [83] could perhaps influence hatching failure rates indirectly, for example, by affecting incubation bout lengths and nest attentiveness in different ways [32, 33, 78].

Contrary to previous work which showed no effects of altitude [20, 39], hatching failure rates in our study were generally lower in eggs incubated at presumably cooler, high-elevation localities. However, the effect of Altitude was marginal before outlier removal and did not vary with Latitude, as we envisioned. This might indicate that factors other than temperature could be involved in shaping altitudinal gradients in hatching failure risks, such as increased parental investment in offspring quality at higher elevations [16, 18].

## Conclusion

Overall, our study provides new insight into the large-scale variation in avian hatching failure rates which we hope will stimulate more research on this important part of avian breeding biology. In addition to confirming some patterns reported earlier, we identify several novel relationships that warrant further investigation. While the proximate mechanisms underlying these relationships are not always clear, some of our key findings are consistent with the notion that thermal constraints may limit avian fecundity at lower latitudes [11, 27]. If so, ongoing changes in global climatic conditions [84] may alter the strength and nature of these constraints, with potential consequences for egg viability and reproductive success. Future research should focus more on hatching failure in tropical birds and aim to disentangle the causes of hatching failure more precisely, particularly by examining the roles of nest microclimate, parental nest attentiveness, and predation risk and how these variables are related to different life history strategies.

## Supplementary Information

Below is the link to the electronic supplementary material.

Supplementary Material 1

Supplementary Material 2

Supplementary Material 3

Supplementary Material 4

## Data Availability

Datasets and code on which the conclusions of the manuscript rely are available in FigShare: 10.6084/m9.figshare.31907512.
