# Supplementary material for: Life history and ecology shape global patterns in avian hatching failure rates
Source: BMC Ecol Evol. 2026 Apr 10;26:35. doi: 10.1186/s12862-026-02515-x (PMC13067641; doi:10.1186/s12862-026-02515-x)
Supplement: Supplementary file 1 — Supplementary Material 1 [file 12862_2026_2515_MOESM1_ESM.docx]

**Life history and ecology shape global patterns in avian hatching failure rates**

**Supplementary material**

**Table S1.** Medians (with interquartile ranges) for continuous variables, along with sample sizes (complete dataset/reduced dataset used in regression models), for all explanatory variables included in the study.

| **Continuous variables** | **Median (interquartile range)** | ***n*** |
| --- | --- | --- |
| Latitude | 42.2^o^ (30.7-52.5^o^) | 516/496 |
| Altitude | 164 m a.s.l. (30.5-396 m a.s.l.) | 515/496 |
| Egg mass | 7.3 g (2.6-31 g) | 494/474 |
| Clutch Size | 3.7 eggs (2.6-4.8 eggs) | 521/496 |
| Generation Length | 3.5 yrs (2.6-5.7 yrs) | 521/496 |
| **Categorical variables** |  |  |
| **Incubation Strategy** |  | 502/496 |
| *Uniparental incubation* | - | 298/296 |
| *Biparental incubation* | - | 204/200 |
| **Nest Type** |  | 521/496 |
| *Open* | - | 337/328 |
| *Closed* | - | 184/168 |
| **Nest Site** |  | 521/496 |
| *Ground* | - | 246/240 |
| *Elevated* | - | 275/256 |

**Table S2.** Candidate beta regression models of hatching failure rates (*n* = 496) explained by Latitude (LAT), Altitude (ALT), Clutch Size (CS), Incubation Strategy (IS), Generation Length (GL), Nest Site (NS), and Nest Type (NT) and interactions between Latitude and Altitude, between Latitude and Clutch Size and between Nest Site and Nest Type. Model performance was evaluated using Akaike Information Criterion (AIC). The models are ordered from smallest to highest ΔAIC values, and models with a ΔAIC < 3 are shown. The best supported model is in bold.

| **Variables** | **K** | **AIC** | **ΔAIC** | **weight** |
| --- | --- | --- | --- | --- |
| LAT^2^ + ALT + CS + IS + GL + NS + CS:LAT^2^ + NT + ALT:LAT^2^ | 14 | -1337.1 | 0.00 | 0.28 |
| LAT^2^ + ALT + CS + IS + GL + NS + CS:LAT^2^ + NT | 12 | -1336.7 | 0.41 | 0.23 |
| LAT^2^ + ALT + CS + IS + GL + NS + CS:LAT^2^ + ALT:LAT^2^ | 13 | -1335.9 | 1.17 | 0.16 |
| LAT^2^ + ALT + CS + IS + GL + NS + CS:LAT^2^ + NT + ALT:LAT^2^ + NS:NT | 15 | -1335.3 | 1.75 | 0.12 |
| **LAT^2^ + ALT + CS + IS + GL + NS + CS:LAT^2^** | **11** | **-1335.3** | **1.78** | **0.12** |
| LAT^2^ + ALT + CS + IS + GL + NS + CS:LAT^2^ + NT + NS:NT | 13 | -1334.8 | 2.23 | 0.09 |

**Table S3.** Candidate beta regression models of hatching failure rates (*n* = 469) explained by Latitude (LAT), Altitude (ALT), Clutch Size (CS), Incubation Strategy (IS), Generation Length (GL), Nest Site (NS), and Nest Type (NT) and interactions between Latitude and Altitude, between Latitude and Clutch Size and between Nest Site and Nest Type. Highly influential points are removed. Model performance was evaluated using Akaike Information Criterion (AIC). The models are ordered from smallest to highest ΔAIC values, and models with a ΔAIC < 3 are shown. The best supported model is in bold.

| **Variables** | **K** | **AIC** | **ΔAIC** | **weight** |
| --- | --- | --- | --- | --- |
| LAT^2^ + ALT + CS + IS + GL + NS + CS:LAT^2^ + NT | 12 | -1397.5 | 0.00 | 0.35 |
| **LAT^2^ + ALT + CS + IS + GL + NS + CS:LAT^2^** | **11** | **-1397.0** | **0.52** | **0.27** |
| LAT^2^ + ALT + CS + IS + GL + NS + CS:LAT^2^ + NT + ALT:LAT^2^ | 14 | -1395.8 | 1.69 | 0.15 |
| LAT^2^ + ALT + CS + IS + GL + NS + CS:LAT^2^ + NT + NS:NT | 14 | -1395.5 | 2.04 | 0.13 |
| LAT^2^ + ALT + CS + IS + GL + NS + CS:LAT^2^ + ALT:LAT^2^ | 13 | -1395.2 | 2.31 | 0.11 |

**Table S4.** Candidate beta regression models of hatching failure rates (*n* = 474) explained by Latitude (LAT), Altitude (ALT), Clutch Size (CS), Incubation Strategy (IS), Generation Length (GL), egg mass (EM), Nest Site (NS), Nest Type (NT) and interactions between Latitude and Altitude, Latitude and Clutch Size and between Nest Site and Nest Type. Model performance was evaluated using Akaike Information Criterion (AIC). The models are ordered from smallest to highest ΔAIC values, and models with a ΔAIC < 3 are shown. The best supported model is in bold.

| **Variables** | **K** | **AIC** | **ΔAIC** | **weight** |
| --- | --- | --- | --- | --- |
| LAT^2^ + ALT + CS + IS + GL + NS + CS:LAT^2^ + ALT:LAT^2^ + NT | 14 | -1327.8 | 0.00 | 0.30 |
| **LAT^2^ + ALT + CS + IS + GL + NS + CS:LAT^2^ + ALT:LAT^2^** | **13** | **-1327.3** | **0.42** | **0.24** |
| LAT^2^ + ALT + CS + IS + GL + NS + CS:LAT^2^ + ALT:LAT^2^ + EM | 14 | -1326.5 | 1.23 | 0.16 |
| LAT^2^ + ALT + CS + IS + GL + NS + CS:LAT^2^ + ALT:LAT^2^ + NT + EM | 15 | -1326.2 | 1.55 | 0.14 |
| LAT^2^ + ALT + CS + IS + GL + NS + CS:LAT^2^ + ALT:LAT^2^ + NT + NS:NT | 16 | -1325.7 | 2.05 | 0.11 |

**Table S5.** Candidate beta regression models of hatching failure rates (*n* = 447) explained by Latitude (LAT), Altitude (ALT), Clutch Size (CS), Incubation Strategy (IS), Generation Length (GL), egg mass (EM), Nest Site (NS), Nest Type (NT) and interactions between Latitude and Altitude, Latitude and Clutch Size and between Nest Site and Nest Type. Highly influential points are removed. Model performance was evaluated using Akaike Information Criterion (AIC). The models are ordered from smallest to highest ΔAIC values, and models with a ΔAIC < 3 are shown. The best supported model is in bold.

| **Variables** | **K** | **AIC** | **ΔAIC** | **weight** |
| --- | --- | --- | --- | --- |
| LAT^2^ + ALT + CS + IS + GL + NS + CS:LAT^2^ + ALT:LAT^2^ | 13 | -1352.0 | 0.00 | 0.31 |
| LAT^2^ + ALT + CS + IS + GL + NS + CS:LAT^2^ + ALT:LAT^2^ + NT | 14 | -1351.5 | 0.51 | 0.24 |
| **LAT^2^ + ALT + CS + IS + GL + NS + CS:LAT^2^** | **11** | **-1350.4** | **1.67** | **0.14** |
| LAT^2^ + ALT + CS + IS + GL + NS + CS:LAT^2^ + NT | 12 | -1350.0 | 2.03 | 0.11 |
| LAT^2^ + ALT + CS + IS + GL + NS + CS:LAT^2^ + ALT:LAT^2^ + EM | 14 | -1350.0 | 2.08 | 0.11 |
| LAT^2^ + ALT + CS + IS + GL + NS + CS:LAT^2^ + ALT:LAT^2^ + NT + NS:NT | 16 | -1349.5 | 2.56 | 0.09 |


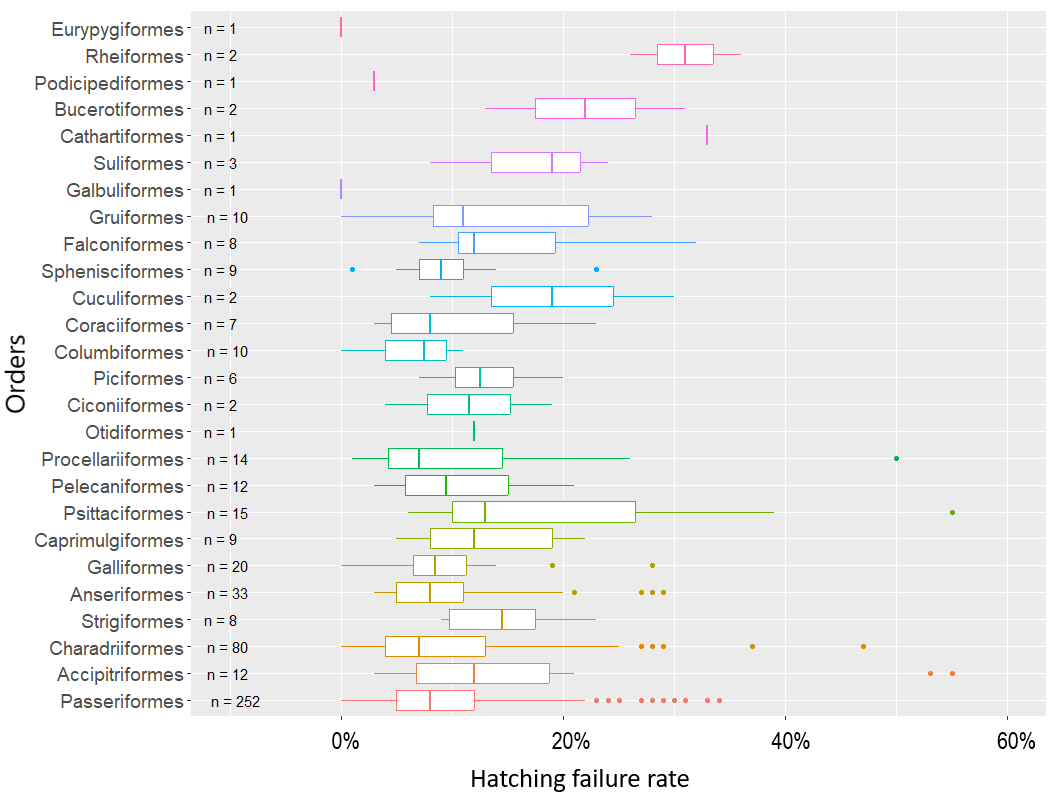


**Fig. S1** Hatching failure rates per phylogenetic order in our dataset. Box plots indicate the median (interior line), interquartile range (boxes), minima and maxima (whiskers) and outliers (dots). Sample sizes per order are shown on the left margin of the plot.


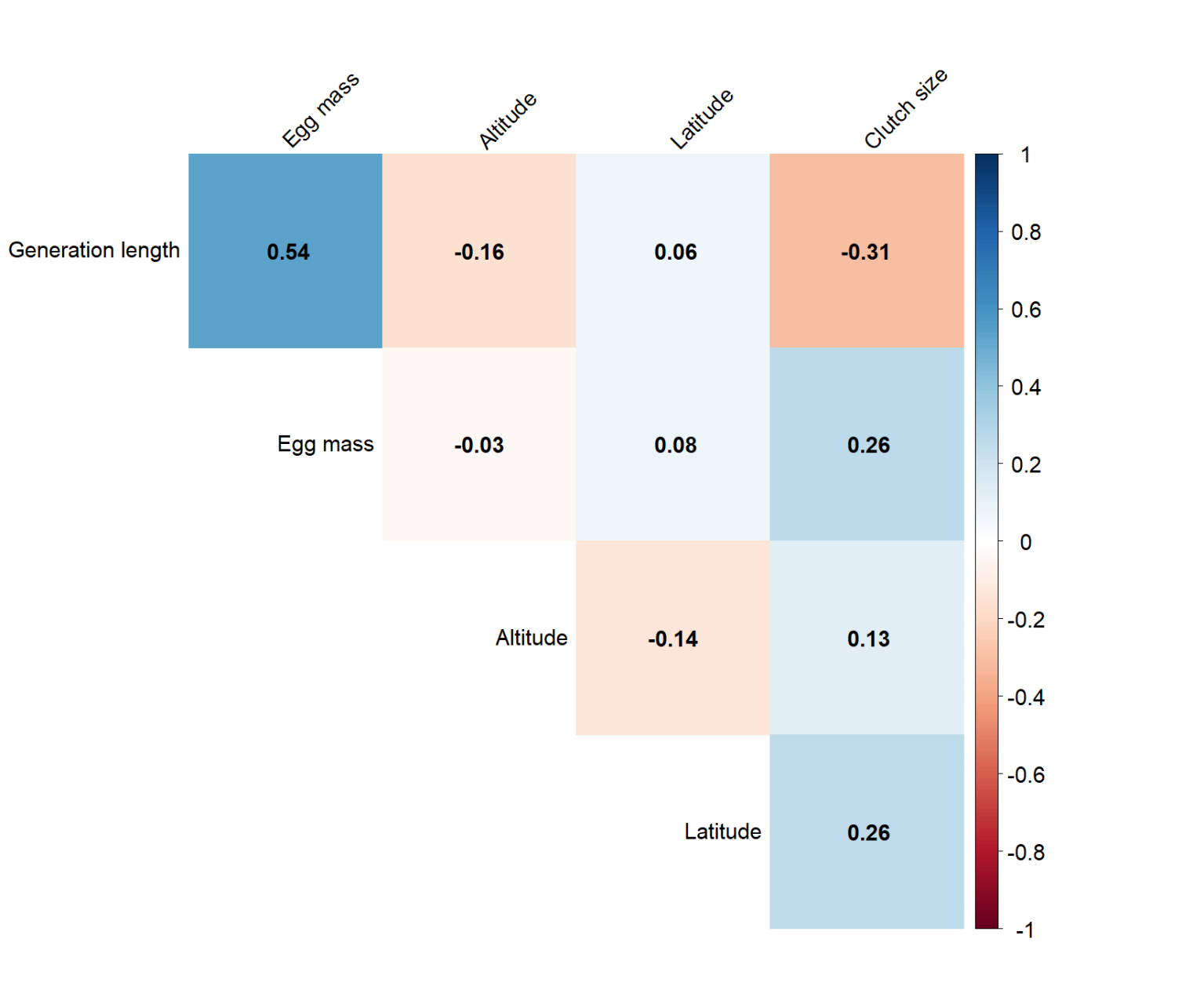


**Fig. S2** Correlation matrix showing the correlations between continuous variables included in the beta regression model shown in Table 2. The numbers reflect correlation coefficients, and the colours visualize the magnitude and direction of the relationships.


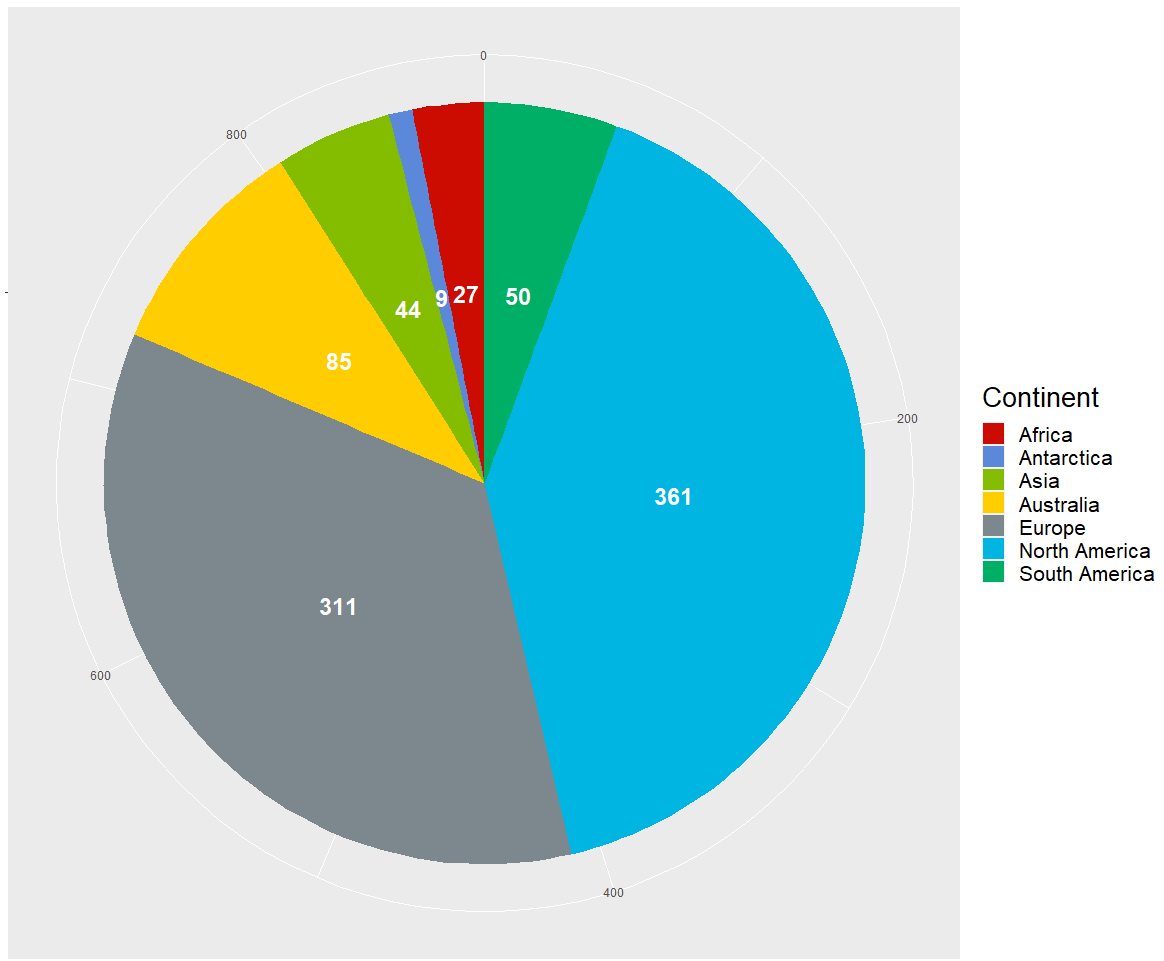


**Fig. S3** Continental distribution of estimates of hatching failure rates included in our dataset.
