# Supplementary figures and images for: Life history and ecology shape global patterns in avian hatching failure rates
Source: BMC Ecol Evol. 2026 Apr 10;26:35. doi: 10.1186/s12862-026-02515-x (PMC13067641; doi:10.1186/s12862-026-02515-x)

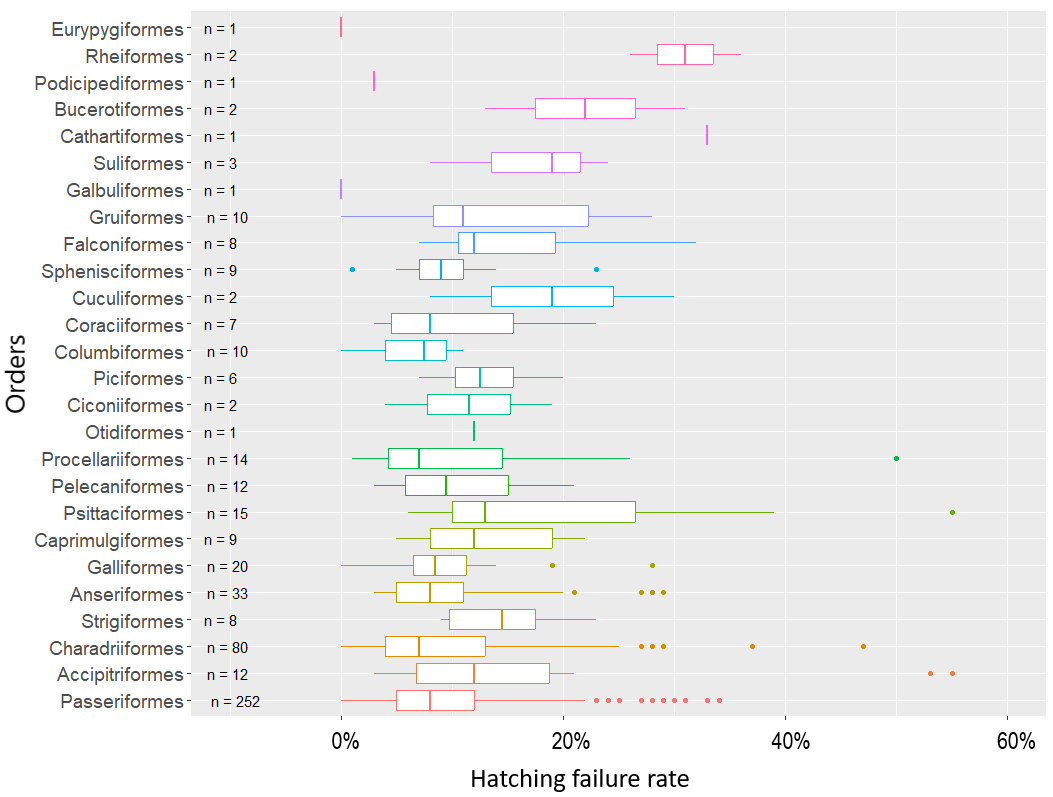

Supplement: Supplementary file 2 — Supplementary Material 2 [file 12862_2026_2515_MOESM2_ESM.tiff]

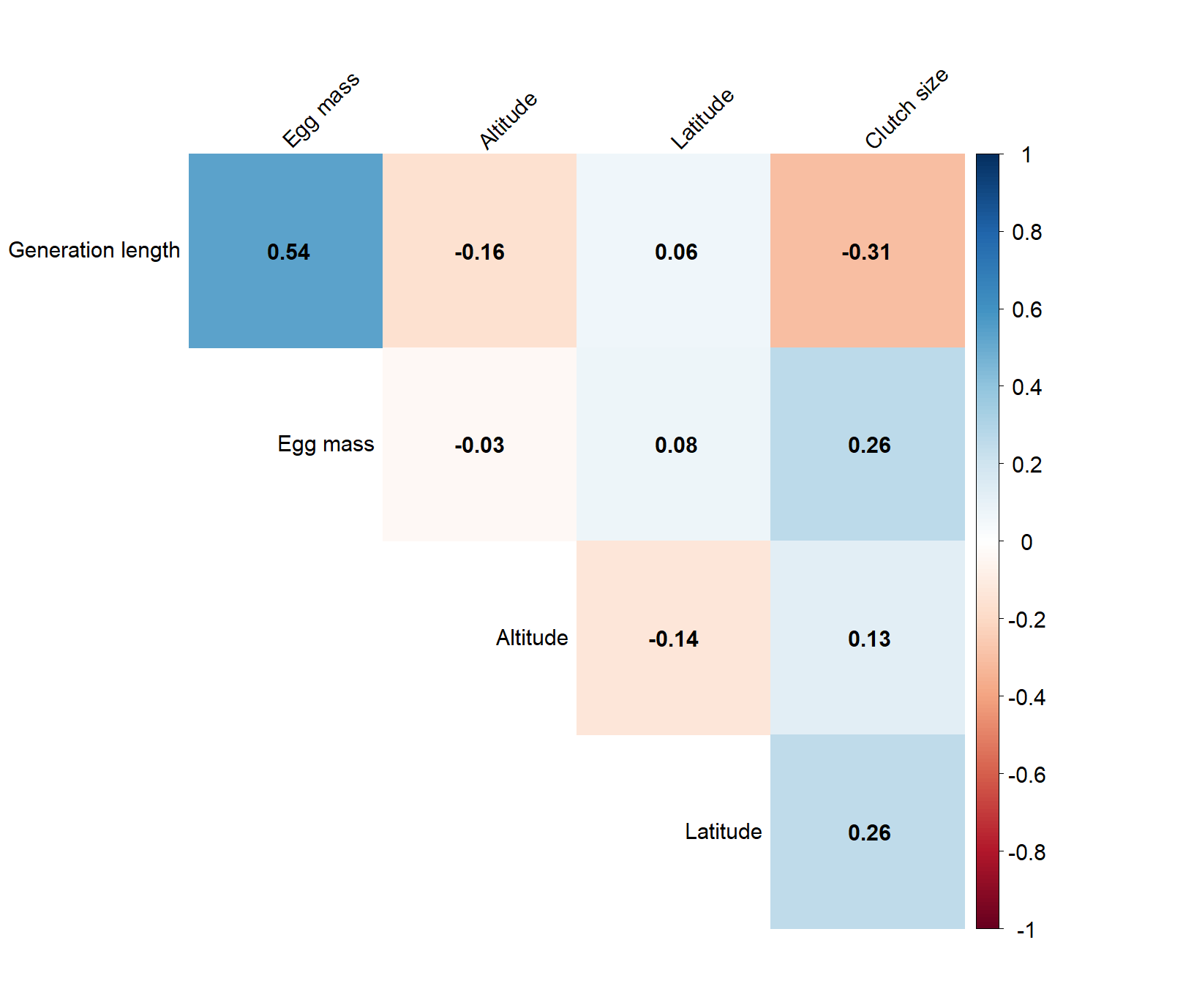

Supplement: Supplementary file 3 — Supplementary Material 3 [file 12862_2026_2515_MOESM3_ESM.tiff]

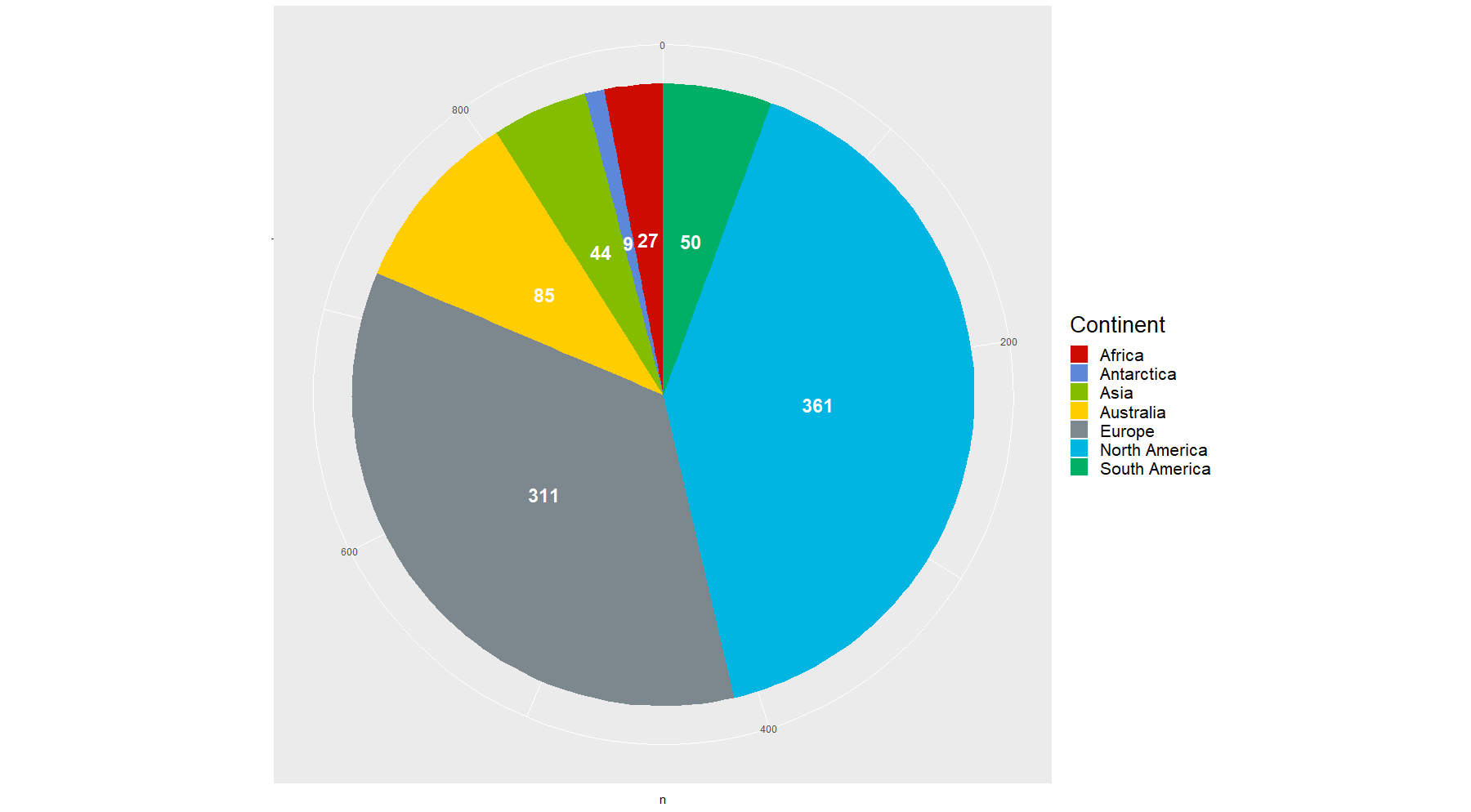

Supplement: Supplementary file 4 — Supplementary Material 4 [file 12862_2026_2515_MOESM4_ESM.tiff]
